# Frequent coexistence of early repolarization pattern, J‐point elevation, and high Sokolow‐Lyon voltage in young men

**DOI:** 10.1002/joa3.12813

**Published:** 2023-01-09

**Authors:** Nagomi Saito, Daigo Nagahara, Naoyuki Kamiyama, Takefumi Fujito, Masayuki Koyama, Atsushi Mochizuki, Toshiyuki Yano, Satoshi Takahashi

**Affiliations:** ^1^ Division of Laboratory Medicine Sapporo Medical University Hospital Sapporo Japan; ^2^ Department of Cardiology Teine Keijinkai Hospital Sapporo Japan; ^3^ Department of Cardiovascular, Renal and Metabolic Medicine Sapporo Medical University School of Medicine Sapporo Japan; ^4^ Department of Public Health Sapporo Medical University School of Medicine Sapporo Japan; ^5^ Department of Infection Control and Laboratory Medicine Sapporo Medical University School of Medicine Sapporo Japan

**Keywords:** androgen, early repolarization, electrocardiogram, J wave, Sokolow‐Lyon voltage

## Abstract

**Background:**

Earlier studies have shown male dominance of an early repolarization (ER) pattern and frequent coexistence with high Sokolow‐Lyon voltage. Although possible involvement of androgen is speculated, the underlying mechanism has not been clarified yet. Previous studies were conducted in adult populations or only in children, and there has been no study in which the ER pattern was investigated in a series of individuals ranging from children before puberty to adults.

**Methods:**

We included 600 individuals comprising six groups according to age: 10–14 years old, 15–19 years old, twenties, thirties, forties, and fifties. Each group had 50 males and 50 females. The distribution of an ER pattern and related ECG parameters were assessed by age and gender.

**Results:**

In early teenagers, there was no significant gender difference in the prevalence of an ER pattern (24% in men vs. 28% in women, *p* = .82). The prevalence of an ER pattern increased after puberty and reached a peak in men in their twenties (42%). With further advance of age, the prevalence of an ER pattern decreased. On the other hand, the prevalence of an ER pattern in women peaked at 28% in teenagers, and it decreased through twenties (20%) to thirties (10%). Similar male dominance after puberty was observed in Sokolow‐Lyon voltage and J‐point elevation but not in P‐wave amplitude.

**Conclusion:**

The prevalence of an ER pattern, Sokolow‐Lyon voltage, and J‐point elevation are all augmented after puberty and decrease with aging, leading to frequent coexistence of these ECG findings in young men.

## Graphical abstract

Earlier studies have shown male dominance of an early repolarization pattern and frequent coexistence with high Sokolow‐Lyon voltage, but the mechanism has not been clarified yet. The male dominance of the prevalence of an ER pattern, Sokolow‐Lyon voltage, and J‐point elevation are all augmented after puberty and decrease with aging, leading to frequent coexistence of these ECG findings in young men.

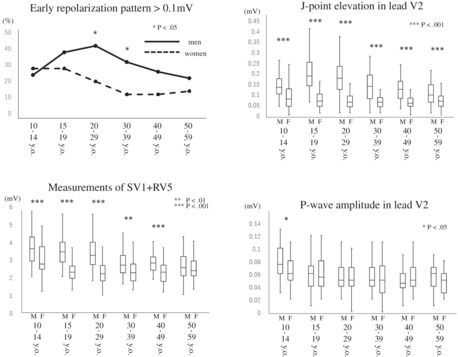

## 1 INTRODUCTION

An early repolarization (ER) pattern is characterized by an elevation of the QRS‐ST junction and QRS notching or slurring pattern in multiple leads, and it was regarded as a benign finding; however, an association between the ER pattern and lethal ventricular arrhythmia was reported in patients with idiopathic ventricular fibrillation^1^ and in a general population.^2^ Earlier studies have shown male dominance of the ER pattern,^2^, ^3^, ^4^, ^5^, ^6^ suggesting possible involvement of androgen. However, most of the previous studies were conducted in adult populations^3^, ^5^, ^6^ or only in children,^7^ and there has been no study in which the ER pattern was investigated in a series of individuals including children before puberty, juveniles, and adults in which the period androgen activity fluctuates widely.^8^

An ER pattern is known to be frequent in individuals with high Sokolow‐Lyon voltage,^2^, ^3^, ^9^ but the underlying mechanism has not been clarified yet. We recently reported that androgen replacement therapy for gender dysphoria induced multiple changes in a 12‐lead electrocardiogram (ECG) including J‐point elevation, augmentation of QRS amplitude, and development of an ER pattern.^10^ Ezaki et al. showed that J‐point elevation in lead V2 in female subjects remained almost constant at all ages but that the J‐point in male subjects was significantly elevated at young ages. They also found that the J‐point elevation was reduced by androgen‐deprivation therapy for prostate carcinoma,^11^ indicating an association between J‐point elevation and androgen activity. Taken together, it is probable that an ER pattern, augmentation of QRS amplitude, and J‐point elevation are often related to androgen activity. The aim of the study was to clarify the prevalence and distribution of an ER pattern by age and gender in relation to ECG parameters such as J‐point elevation and Sokolow‐Lyon voltage in a wide range of individuals including children before puberty to adults.

## 2 METHODS

### 2.1 Study population

The study population consisted of 600 individuals comprising six groups according to age: 10–14 years old, 15–19 years old, twenties, thirties, forties, and fifties. Each group had 50 males and 50 females. They were enrolled from outpatients who underwent a standard 12‐lead ECG test and had normal sinus rhythm at Sapporo Medical University Hospital between January 2017 and March 2020 after exclusion of subjects who met the following exclusion criteria: individuals with organic heart disease, congenital disease, pectus excavatum, hypertension, diabetes mellitus, collagen disease, malignancy, or epilepsy, individuals taking antipsychotic drugs, individuals with malnutrition (serum albumin <3.5 g/dl), anemia (hemoglobin <13.1 g/dl in males and <12.1 g/dl in females), abnormal ECG (conduction abnormality, QRS width more than 120 ms, significant ST deviation, Brugada ECG pattern, atrial fibrillation/flutter on the ECG, paced rhythm, and frequent premature beat), sinus tachycardia (heart rate >100 beats per minute), or sinus bradycardia (heart rate <50 beats per minute), and individuals with the presence of significant noise precluding evaluation of an ER pattern. We included consecutive subjects until each age and gender group was filled with 50 subjects. Once a certain group was filled with 50 subjects, subsequent subjects who were supposed to be categorized to the same group were skipped for analysis, leading to total assessment of 3254 subjects until all groups were filled with 50 subjects. We excluded individuals with structural heart disease by a past history in a medical record and abnormal electrocardiogram. Routine use of transthoracic echocardiography for the detection of organic heart disease was not incorporated in the protocol of the present study. We assessed the prevalence and distribution of an ER pattern and its association with ECG parameters in different age groups of men and women.

### 2.2 Analysis of 12‐lead ECG

A standard 12‐lead ECG was recorded using FCP‐8800 or FX‐7542 (Fukuda Denshi Co., Ltd.) for 20 s at a speed of 25 mm/s, a sampling rate of 8 kHz, and a bandwidth of 0.25–35 Hz. Off‐line ECG measurements were performed using an automatic digital ECG analysis program equipped with the electrocardiographs, and all ECGs were manually checked by an experienced physician. An ER pattern was defined as a J‐point elevation of >0.1 mV with a notch or slur configuration in two contiguous leads in leads I, aVL, II, III, aVF, and V4‐V6. In subjects with a slurring ER pattern, J‐point amplitude was measured at the initial portion of the slurring that starts to diverge from the downstroke of the R wave by more than 10 degrees in angle. In subjects with a notched ER pattern, J‐point amplitude was measured at the peak deflection of the J wave.^12^ The sum of amplitude of S wave in lead V1 and amplitude of R wave in lead V5 was measured as the Sokolow‐Lyon voltage.^13^

### 2.3 Statistical analysis

Statistical values are shown as means ± 1 SD for normally distributed variables and as median values with interquartile ranges (IQR) for non‐normally distributed variables. The significance of differences in continuous variables between two groups was assessed using the t‐test for normally distributed variables and the Mann–Whitney *U* test for non‐normally distributed variables. Fisher's exact test was used to compare prevalences. Statistical significance was set at *p* < .05. Binominal logistic regression analysis was used to identify independent predictors of ER pattern. The analyses were performed using JMP software (version 15.1.0, SAS Institute).

## 3 RESULTS

### 3.1 Comparison of clinical backgrounds and ECG parameters in men and women

A comparison of clinical backgrounds and parameters of 12‐lead ECG in men and women is shown in Table 1. Ages (median [IQR] 29.5 [17–45] years old vs. 29.5 [17–44] years old, *p* = .94) were comparable in the two groups. Body mass index (median [IQR] 22.2 [19.7–24.6] kg/m^2^ vs. 20.7 [18.7–23.0] kg/m^2^, *p* < .001) was significantly higher and the heart rate (median [IQR] 65 [58–72] beats per minute vs. 66 [60–74] beats per minute, *p* = .007) was significantly lower in men than in women. An ER pattern of more than 0.1 mV was observed in 24.7% of the study subjects and the prevalence was significantly higher in men than in women (30.7% vs. 18.7%, *p* < .001). An ER pattern of more than 0.2 mV was observed in 6.5% of the study subjects with male dominance (10.7% vs. 2.3%, *p* < .001). The Sokolow‐Lyon voltage (median [IQR] 3.01 [2.45–3.65] mV vs. 2.35 [1.98–2.81] mV, *p* < .001) and R wave amplitude in lead II (median [IQR] 1.23 [0.94–1.56] mV vs. 1.05 [0.82–1.34] mV, *p* < .001) were significantly higher in men than in women, but there was no significant difference between men and women in P‐wave amplitudes in lead I (median [IQR] 0.06 [0.04–0.07] mV vs. 0.06 [0.05–0.07] mV, *p* = .99), lead II (median [IQR] 0.10 [0.07–0.13] mV vs. 0.10 [0.07–0.13] mV, *p* = .32), or lead V2 (median [IQR] 0.06 [0.04–0.07] mV vs. 0.05 [0.04–0.07] mV, *p* = .38). J‐point elevations in lead V2 (median [IQR] 0.14 [0.09–0.19] mV vs. 0.06 [0.04–0.09] mV, *p* < .001) and V3 (median [IQR] 0.11 [0.06–0.17] mV vs. 0.04 [0.02–0.06] mV, *p* < .001) and T‐wave amplitudes in lead V2 (median [IQR] 0.56 [0.37–0.79] mV vs. 0.30 [0.20–0.43] mV, *p* < .001) and V3 (median [IQR] 0.68 [0.48–0.86] mV vs. 0.42 [0.28–0.55] mV, *p* < .001) were significantly higher in men than in women. Bazett's corrected QT interval was significantly shorter in men than in women (0.400 ± 0.020 s vs. 0.413 ± 0.020 s, *p* < .001).

**TABLE 1:** Clinical characteristics of the study subjects

Variables	All subjects	Men	Women	*p* value
	*n* = 600	*n* = 300	*n* = 300	
Age (years)	29.5 [17–45]	29.5 [17–45]	29.5 [17–44]	.94
Height (cm)	162 [155–170]	170 [165–174]	157 [153–161]	<.001*
Weight (kg)	57 [49–66]	64 [56–72]	51 [45–58]	<.001*
Body mass index (kg/m^2^)	21.4 [19.2–23.9]	22.2 [19.7–24.6]	20.7 [18.7–23.0]	<.001*
Heart rate (bpm)	65.5 [59–73]	65 [58–72]	66 [60–74]	.007*
ER pattern (%), *n*				
>0.1 mV	24.7 (148)	30.7 (92)	18.7 (56)	<.001*
>0.2 mV	6.5 (39)	10.7 (32)	2.3 (7)	<.001*
J point elevation (mV)				
In V2	0.09 [0.05–0.15]	0.14 [0.09–0.19]	0.06 [0.04–0.09]	<.001*
In V3	0.06 [0.03–0.12]	0.11 [0.06–0.17]	0.04 [0.02–0.06]	<.001*
T amplitude (mV)				
In V2	0.41 [0.25–0.62]	0.56 [0.37–0.79]	0.30 [0.20–0.43]	<.001*
In V3	0.52 [0.36–0.74]	0.68 [0.48–0.86]	0.42 [0.28–0.55]	<.001*
P amplitude (mV)				
In I	0.06 [0.04–0.07]	0.06 [0.04–0.07]	0.06 [0.05–0.07]	.99
In II	0.10 [0.07–0.13]	0.10 [0.07–0.13]	0.10 [0.07–0.13]	.32
In V2	0.05 [0.04–0.07]	0.06 [0.04–0.07]	0.05 [0.04–0.07]	.38
In V3	0.06 [0.04–0.07]	0.06 [0.05–0.07]	0.05 [0.04–0.07]	.01*
QRS amplitude (mV)				
In II	1.13 [0.87–1.46]	1.23 [0.94–1.56]	1.05 [0.82–1.34]	<.001*
S amplitude (mV)				
In V1	1.05 [0.75–1.39]	1.19 [0.88–1.49]	0.98 [0.69–1.24]	<.001*
R amplitude (mV)				
In V5	1.59 [1.26–1.96]	1.83 [1.48–2.20]	1.39 [1.13–1.67]	<.001*
SV1 + RV5	2.63 [2.19–3.29]	3.01 [2.45–3.65]	2.35 [1.98–2.81]	<.001*
Transitional zone	4 [3–4]	4 [4–4]	4 [3–4]	.18
U amplitude (mV)				
In V3	0.04 [0.03–0.05]	0.04 [0.03–0.05]	0.04 [0.03–0.05]	.82
Corrected QT interval	0.41 ± 0.02	0.40 ± 0.02	0.41 ± 0.02	<.001*

*Note*: Data are presented as the mean value ± SD for normally distributed variables, the median [interquartile range] for non‐normally distributed variables, or as the percentage (number) of subjects.

Abbreviation: ER, early repolarization.

* *p* < .05.

### 3.2 Prevalences and distributions of an ER pattern and ECG measurements in different age groups of men and women

#### 3.2.1 Prevalence and distribution of an ER pattern

**FIGURE 1:**
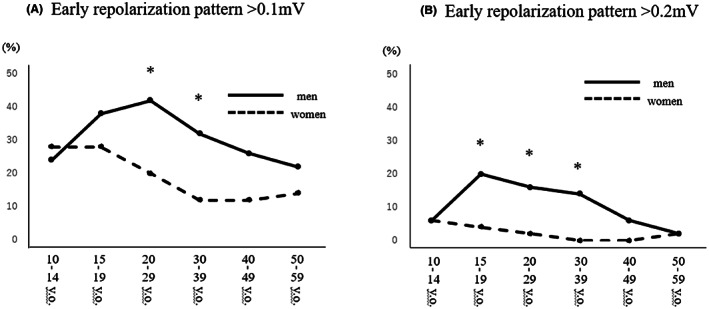
Distribution of early repolarization patterns >0.1 mV (A) and >0.2 mV (B) in different age groups of men and women. *p < .05

The prevalences of an ER pattern of more than 0.1 mV according to age and gender are shown in Figure 1A. In early teenagers, there was no significant difference between the prevalences of an ER pattern in men and women (24% vs. 28%, *p* = .82). The prevalence of an ER pattern increased after puberty and reached a peak in men in their twenties (42%). With further advance of age, the prevalence of an ER pattern decreased. In contrast, the prevalence of an ER pattern in women peaked at the 28% in early teenagers and late teenagers and decreased through the twenties (20%) to thirties (10%). Therefore, the prevalences of an ER pattern were significantly higher in men than in women in their twenties (42% vs. 20%, *p* = .03) and thirties (32% vs. 10%, *p* = .01). The prevalences of an ER pattern of more than 0.2 mV showed a similar distribution except for the peak being in late teenagers in men. There was a significant male dominance in the prevalences of an ER pattern in late teenagers (20% vs. 4%, *p* = .03), subjects in their twenties (16% vs. 2%, *p* = .03), and subjects in their thirties (14% vs. 0%, *p* = .01) (Figure 1B). The type of an ER pattern (notch or slur) and the location (inferior, lateral, or both) are shown in Table 2. The prevalences of the notch type in all subjects were significantly higher in men than in women (77.2% vs. 57.9%, *p* = .02).

**TABLE 2:** Type of an ER pattern

Age groups	10–14 years old	15–19 years old	20–29 years old	30–39 years old	40–49 years old	50–59 years old	Total
Men							
Notch/slur (%)	83.3/16.7	84.2/15.8	66.7/33.3	68.8/31.3	76.9/23.1	90.9/9.1	77.2/22.8*
Inferior/lateral/both (%)	41.7/25.2/33.3	21.1/15.8/63.2	42.9/23.8/33.3	37.5/31.3/31.3	69.2/15.4/15.4	81.8/9.1/9.1	45.7/20.7/33.7
Women							
Notch/slur (%)	73.3/26.7	57.1/42.9	40.0/60.0	80.0/20.0	33.3/66.7	57.1/42.9	57.9/42.1*
Inferior/lateral/both (%)	35.7/21.4/42.8	57.1/21.4/21.4	50.0/20.0/30.0	60.0/0.0/40.0	66.7/0.0/33.3	42.9/0.0/57.1	50.0/14.3/35.7

Abbreviation: ER, early repolarization.

* *p* < .05.

#### 3.2.2 Measurements and distributions of ECG parameters

**FIGURE 2:**
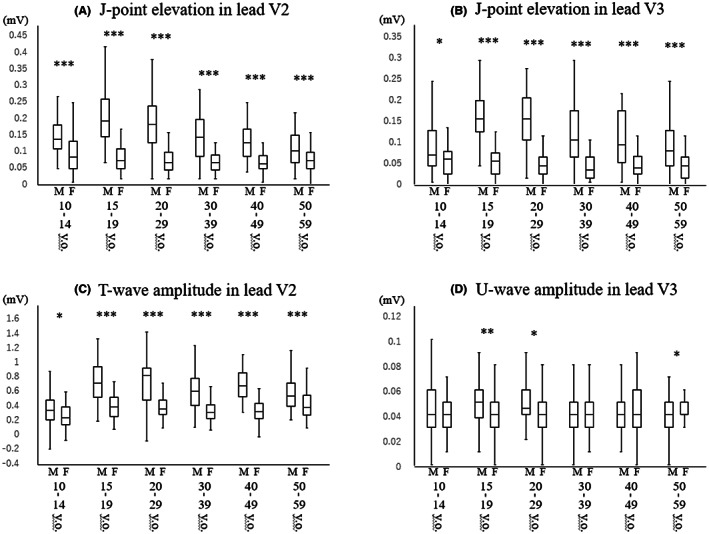
Distribution of J‐point elevations in lead V2 (A) and lead V3 (B), T‐wave amplitude in lead V2 (C), and U‐wave amplitude in lead V3 (D) in different age groups of men and women. *p < .05; **p < .01; ***p < .001

**FIGURE 3:**
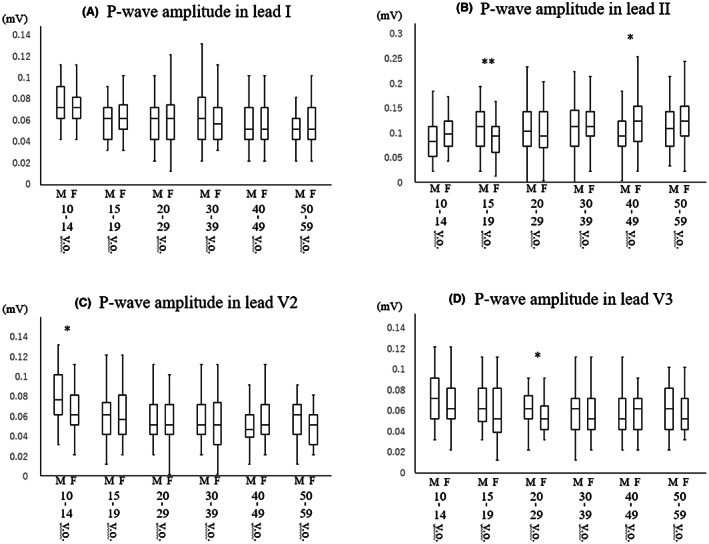
Distribution of P‐wave amplitude in lead I (A), lead II (B), lead V2 (C), and lead V3 (D) in different age groups of men and women. *p < .05; **p < .01; ***p < .001.

**FIGURE 4:**
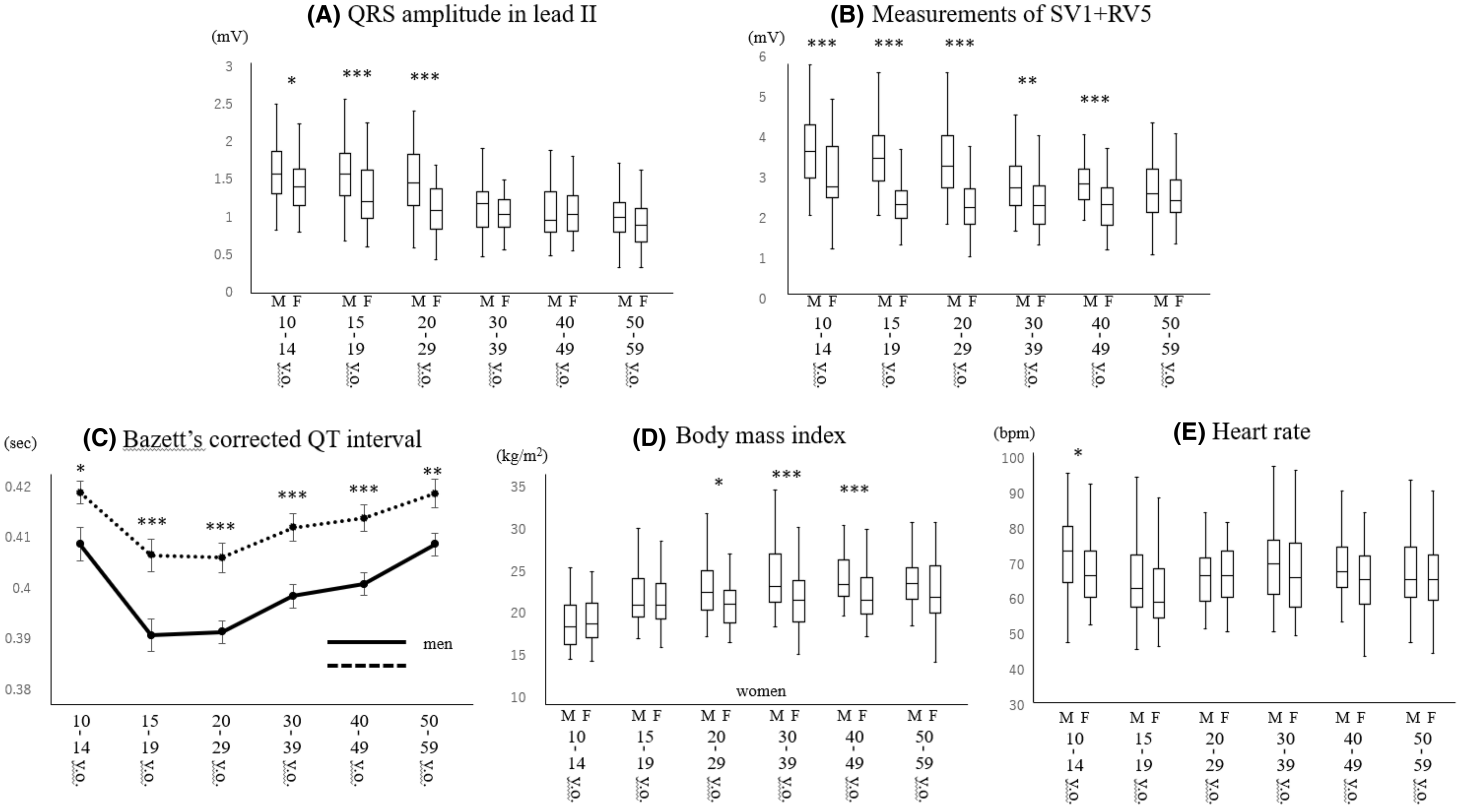
Distribution of QRS amplitude in lead II (A), measurement of SV1 + RV5 (B), Bazett's corrected QT interval (C), body mass index (D), and heart rate (E) in different age groups of men and women. *p < .05; **p < .01; ***p < .001

J‐point elevations in leads V2 and V3, T‐wave amplitude in lead V2, and U‐wave amplitude in lead V3 in different age groups of men and women are shown in Figure 2A–D. J‐point elevations in lead V2 and V3 were significantly higher in men than in women at all ages, but male dominance was prominent especially in late teenagers (V2: median [IQR] 0.19 [0.14–0.25] mV vs. 0.07 [0.04–0.10] mV, *p* < .001) and subjects in their twenties (V2: median [IQR] 0.18 [0.12–0.23] mV vs. 0.06 [0.04–0.09] mV, *p* < .001). T‐wave amplitudes were significantly higher in men than in women at all ages, but male dominance became prominent after early teens. Male dominance in U‐wave amplitude in lead V3 was observed in late teenagers (median [IQR] 0.05 [0.04–0.06] mV vs. 0.04 [0.03–0.05] mV, *p* = .006) and subjects in their twenties (median [IQR] 0.05 [0.04–0.06] mV vs. 0.04 [0.03–0.05] mV, *p* = .04). P‐wave amplitudes in leads I, II, V2, and V3 in different age and gender groups are shown in Figure 3A–D. Male dominance was not observed in P‐wave amplitudes except in lead II in late teenagers (median [IQR] 0.11 [0.07–0.14] mV vs. 0.09 [0.06–0.11] mV, *p* = .009), in lead V2 in early teenagers (median [IQR] 0.08 [0.06–0.10] mV vs. 0.06 [0.05–0.08] mV, *p* = .03), and in lead V3 in subjects in their twenties (median [IQR] 0.06 [0.05–0.07] mV vs. 0.05 [0.04–0.06] mV, *p* = .03). QRS amplitude in lead II, Sokolow‐Lyon voltage, corrected QT interval, body mass index, and heart rate in different age groups of men and women are shown in Figure 4A–E. QRS amplitude in lead II was greater in men than in women in early teenagers, and male dominance became more prominent in late teenagers (median [IQR] 1.53 [1.26–1.81] mV vs. 1.16 [0.95–1.58] mV, *p* < .001) and subjects in their twenties (median [IQR] 1.42 [1.23–1.78] mV vs. 1.05 [0.81–1.33] mV, *p* < .001). Sokolow‐Lyon voltage was significantly higher in men than in women at all ages except fifties, and the gender difference was prominent in late teenagers (median [IQR] 3.43 [2.87–3.92] mV vs. 2.28 [1.98–2.60] mV, *p* < .001) and subjects in their twenties (median [IQR] 3.23 [2.71–3.97] mV vs. 2.19 [1.82–2.62] mV, *p* < .001). Corrected QT interval was significantly shorter in men than in women at all ages, but the gender gap was prominent in late teenagers (0.391 ± 0.023 s vs. 0.407 ± 0.022 s, *p* < .001) and subjects in their twenties (0.391 ± 0.016 s vs. 0.406 ± 0.020 s, *p* < .001). Thus, the male dominance in Sokolow‐Lyon voltage and J‐point elevation was prominent in late teenagers and subjects in their twenties and diminished with advance of age as in the case of an ER pattern of more than 0.2 mV. On the other hand, this type of male dominance after puberty was not observed in P‐wave amplitude and body mass index.

### 3.3 Representative ECGs

**FIGURE 5:**
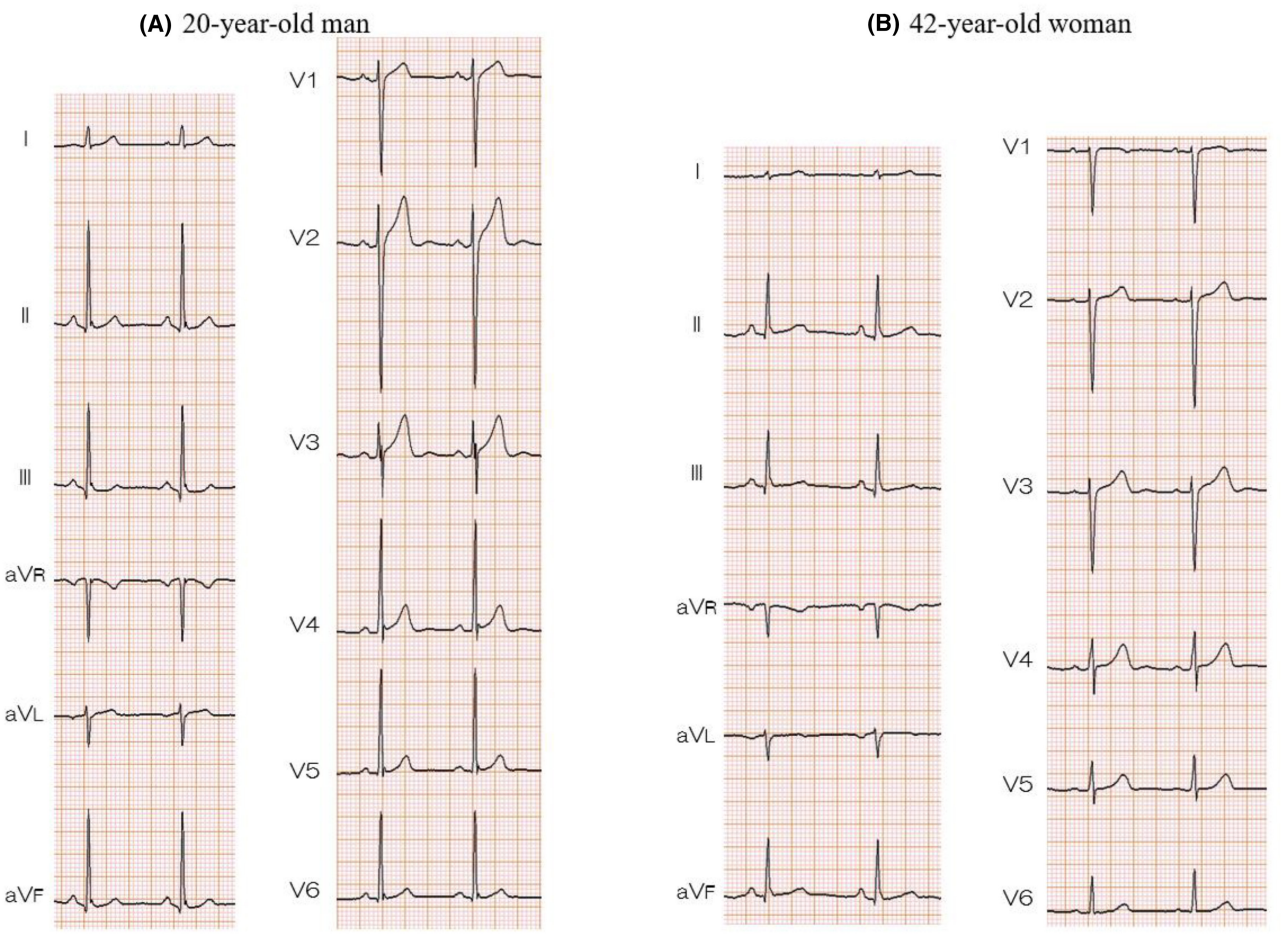
A 20‐year‐old man (A) showed an ER pattern of more than 0.1 mV, high Sokolow‐Lyon voltage, and J‐point elevation in lead V2. The ECG from a 42‐year‐old woman (B) showed an ER pattern of more than 0.1 mV and normal Sokolow‐Lyon voltage without J‐point elevation

Representative ECGs are shown in Figure 5. A 20‐year‐old man (Figure 5A) showed an ER pattern of more than 0.1 mV, high Sokolow‐Lyon voltage, and J‐point elevation in lead V2. The ECG from a 42‐year‐old woman (Figure 5B) showed an ER pattern of more than 0.1 mV and normal Sokolow‐Lyon voltage without J‐point elevation.

### 3.4 ECG parameters associated with ER pattern

The results of multivariate logistic analysis for clarifying explanatory variables of an ER pattern of more than 0.1 mV and an ER pattern of more than 0.2 mV are shown in Tables 3 and 4, respectively. Sokolow‐Lyon voltage was independently associated with the presence of both an ER pattern of more than 0.1 mV and an ER pattern of more than 0.2 mV even after adjustment in model 1 (adjusted for age, gender, and body mass index), model 2 (model 1 + heart rate and corrected QT interval), and model 3 (model 2 + J‐point elevation in lead V2 and T‐wave amplitude in V2).

**TABLE 3:** Multivariate analysis for prediction of early repolarization pattern >0.1 mV

	Odd's ratio	95% CI		*p* value
Unadjusted				
Sokolow‐Lyon voltage	2.06	1.65	2.58	<.001
Adjusted				
Model 1 adjusted for age, gender, BMI	1.95	1.51	2.52	<.001
Model 2 adjusted for model 1 + heart rate + corrected QT interval	2.00	1.54	2.60	<.001
Model 3 adjusted for model 2 + J‐point elevation in V2 + T‐wave amplitude in V2	2.20	1.66	2.92	<.001

**TABLE 4:** Multivariate analysis for prediction of early repolarization pattern >0.2 mV

	Odd's ratio	95% CI		*p* value
Unadjusted				
Sokolow‐Lyon voltage	2.50	1.77	3.54	<.001
Adjusted				
Model 1 adjusted for age, gender, BMI	1.90	1.26	2.85	<.01
Model 2 adjusted for model 1 + heart rate + corrected QT interval	1.94	1.29	2.93	<.01
Model 3 adjusted for model 2 + J‐point elevation in V2 + T‐wave amplitude in V2	2.22	1.41	3.48	<.001

## 4 DISCUSSION

Earlier studies have shown that an ER pattern is frequent in men,^2^, ^3^, ^4^, ^5^, ^6^ younger generation,^2^, ^3^, ^4^, ^5^, ^6^ and individuals with high Sokolow‐Lyon voltage.^2^, ^3^, ^9^ Although androgen is presumed to facilitate the development of an ER pattern, there has been no study in which the ER pattern was evaluated in a series of generations from children before puberty to adults in which the period androgen activity fluctuates widely. Data in previous studies were mostly obtained from limited populations such as adults only,^2^, ^3^, ^5^, ^6^ athletes,^9^ and children.^7^ The present study is the first study in which the prevalence of an ER pattern and its association with ECG parameters were evaluated in a broad population ranging from children before puberty to middle‐aged adults. In the present study, significant and prominent male dominance was observed in the prevalence of an ER pattern, Sokolow‐Lyon voltage, and J‐point elevation, especially in late teenagers and subjects in their twenties. Sokolow‐Lyon voltage was independently associated with an ER pattern.

### 4.1 Mechanisms of the development of an ER pattern and the difference between men and women

Noseworthy et al.^3^ carried out a study in a general population with ages ranging from 30 to 70 years and calculated the prevalences of an ER pattern according to age and gender. The prevalence of an ER pattern was highest at 10.4% in men in their thirties. With further advance of age, the prevalence of an ER pattern decreased and was 0.7% in subjects in their seventies. In contrast, the rates were low regardless of age in women (2.3% in their thirties and 0.7% in their seventies). Maury et al.^5^ showed similar age and gender differences in the prevalence of an ER pattern in a general population (35–44 years old: 27.1% in men vs. 5.7% in women, 45–54 years old: 21.4% in men vs. 4.1% in women, 55–64 years old: 15.1% in men vs. 7.8% in women). In contrast, there was no gender difference in the prevalence of an ER pattern in children with ages ranging from 13 to 18 years (40% in boys vs. 40% in girls).^7^ Although previous studies have shown that an ER pattern is frequent in men and in the younger generation,^3^, ^4^, ^5^, ^6^ the present study showed for the first time that the prevalence of an ER pattern increased from early teenagers to late teenagers and reached a peak in men in their twenties, a period that is known to be associated with high testosterone activity.^8^

It is also known that an ER pattern is frequent in athletes^9^ and in individuals with sinus bradycardia,^2^, ^6^, ^9^ and increased parasympathetic tone is therefore speculated to be involved in the development of an ER pattern. In a study by Abhishekh et al.,^14^ parasympathetic tone was analyzed with heart rate variability in 189 healthy volunteers and they reported that parasympathetic tone diminished with advance of age. In the present study, the prevalence of an ER pattern was highest in teenage females and decreased with aging; however, there was no significant change in heart rate suggesting aging‐related decline in parasympathetic tone. In light of these different peaks in the ER pattern in men and women, different factors might be responsible for the development of an ER pattern.

### 4.2 Factors associated with both Sokolow‐Lyon voltage and ER pattern

In previous studies, an ER pattern was frequently observed in individuals with high Sokolow‐Lyon voltage.^2^, ^3^, ^9^ In the present study, high Sokolow‐Lyon voltage was independently associated with the presence of an ER pattern in multivariate logistic analysis (Tables 3 and 4). Earlier studies showed that an ER pattern is frequent in young men, and the fact that young men have a thin chest wall compared with that in women with breasts or that in overweight middle‐aged men was proposed to be the reason for the strong association between the presence of an ER pattern and high Sokolow‐Lyon voltage.^3^ In the present study, the gender difference in Sokolow‐Lyon voltage was augmented in late teenagers and subjects in their twenties; however, P‐wave amplitude did not show such a gender difference. If the gender gap in Sokolow‐Lyon voltage was the manifestation of a thin chest wall in young male subjects, similar age and gender gaps should also have been observed in P‐wave amplitude. Therefore, it is not reasonable to explain the frequent coexistence of an ER pattern and high Sokolow‐Lyon voltage by body habitus alone.

The possible mechanism for the high Sokolow‐Lyon voltage in males and the relationships between the prevalence of an ER pattern, high Sokolow‐Lyon voltage, and androgen activity have not been clarified yet. Androgen seems to be involved in both the mechanism of androgen‐induced cardiac hypertrophy and the development of an ER pattern. Young males have high androgen activity compared to that in females and older males, leading to frequent coexistence of high Sokolow‐Lyon voltage and the presence of an ER pattern in young males. The findings in earlier studies indicate that androgen induces cardiac hypertrophy and is hence responsible for consequent augmentation of QRS amplitudes. First, an animal experimental model in a previous study showed androgen‐induced cardiac hypertrophy.^15^ Second, in an earlier study, it was found that the QRS amplitude in males significantly increased after puberty, leaving a huge gap between males and females, and then declined with aging, while there was no significant difference between the QRS amplitudes in male and female children before puberty.^16^ Third, androgen replacement therapy for gender dysphoria induced multiple changes in a 12‐lead ECG including J‐point elevation, augmentation of QRS amplitude, and development of an ER pattern.^10^ That study included 13 subjects with gender dysphoria and androgen therapy augmented the mean Sokolow‐Lyon voltage from 2.32 ± 0.71 mV to 3.25 ± 0.75 mV. However, there was no similar change in P‐wave amplitude, suggesting that the change in QRS amplitude was not the result of the change of body habitus caused by androgen‐induced anabolic metabolism. On the other hand, there are also several lines of evidence showing that androgen is responsible for the development of an ER pattern. First, Junttila et al. measured serum testosterone levels in 2755 men and showed that the prevalences of an ER pattern were ~3%, 5%, and 8% in the first, second, and third tertiles of testosterone, respectively.^17^ Second, in several previous studies in which the prevalence of an ER pattern was assessed according to age and gender, the prevalence of an ER pattern was higher in young males than in females and older males.^3^, ^4^, ^5^, ^6^ Third, androgen replacement therapy for gender dysphoria increased the prevalence of an ER pattern from 15.4% to 61.5%.^10^

In a study by Ezaki et al.,^11^ J‐point elevation in lead V2 in female subjects remained almost constant at all ages, but the J‐point was significantly elevated in young male subjects. They also found that the J‐point elevation was reduced by androgen‐deprivation therapy for prostate carcinoma. In a study by Surawicz et al.,^18^ 12‐lead ECG patterns in different age and gender groups were evaluated and it was found that young males have characteristic ECG findings such as J‐point elevation, steeply ascending T‐wave, and augmentation of T‐wave amplitude. Gender differences in these ECG parameters such as the prevalence of an ER pattern, Sokolow‐Lyon voltage, and J‐point elevation, T‐wave amplitude are all augmented after puberty and decrease with aging, leading to frequent coexistence of these ECG findings in young men.

### 4.3 Limitations

This study has some limitations. First, the participants were recruited from patients of our university hospital, not from healthy volunteers or individuals with regular medical checkups. Although we conducted thorough examinations to exclude patients with heart disease or systemic medical problems, the study population may not have been completely healthy individuals. Second, we speculated that an ER pattern, Sokolow‐Lyon voltage, and J‐point elevation were mediated by androgen, but data on testosterone measurements were not available in the present study. Third, since the present study was a cross‐sectional study, a causal relationship between ER pattern and Sokolow‐Lyon voltage is unclear. Moreover, the impact of circadian or day‐to‐day variation of the J‐point amplitude^19^ was not taken into account in the present study.

## 5 CONCLUSION

The prevalence of an ER pattern, Sokolow‐Lyon voltage, and J‐point elevation are all augmented after puberty and decrease with aging, leading to frequent coexistence of these ECG findings in young men.

## CONFLICT OF INTEREST

The authors declare no conflict of interests for this article.

## IRB INFORMATION

The study protocol was approved by the institutional review board of Sapporo Medical University (reference no. 322–239), and it conformed to the provisions of the Declaration of Helsinki.
